# Speeding up training of automated bird recognizers by data reduction of audio features

**DOI:** 10.7717/peerj.8407

**Published:** 2020-01-27

**Authors:** Allan G. de Oliveira, Thiago M. Ventura, Todor D. Ganchev, Lucas N.S. Silva, Marinêz I. Marques, Karl-L. Schuchmann

**Affiliations:** 1 Computational Bioacoustics Research Unit (CO.BRA), National Institute for Science and Technology in Wetlands (INAU), Universidade Federal de Mato Grosso, Cuiabá, Mato Grosso, Brazil; 2 Institute of Computing, Universidade Federal de Mato Grosso, Cuiabá, Mato Grosso, Brazil; 3 Faculty of Computing and Automation, Technical University of Varna, Varna, Bulgaria; 4 Institute of Bioscienses, Universidade Federal de Mato Grosso, Cuiabá, Mato Grosso, Brazil; 5 Postgraduate Program in Ecology and Biodiversity Conservation, Institute of Biosciences, Universidade Federal de Mato Grosso, Cuiabá, Mato Grosso, Brazil; 6 Postgraduate Program in Zoology, Institute of Biosciences, Universidade Federal de Mato Grosso, Cuiabá, Mato Grosso, Brazil; 7 Zoological Research Museum Alexander Koenig and University of Bonn, Bonn, Germany

**Keywords:** Data representation, Data reduction, Random sampling, Uniform sampling, Piecewise aggregate approximation

## Abstract

Automated acoustic recognition of birds is considered an important technology in support of biodiversity monitoring and biodiversity conservation activities. These activities require processing large amounts of soundscape recordings. Typically, recordings are transformed to a number of acoustic features, and a machine learning method is used to build models and recognize the sound events of interest. The main problem is the scalability of data processing, either for developing models or for processing recordings made over long time periods. In those cases, the processing time and resources required might become prohibitive for the average user. To address this problem, we evaluated the applicability of three data reduction methods. These methods were applied to a series of acoustic feature vectors as an additional postprocessing step, which aims to reduce the computational demand during training. The experimental results obtained using Mel-frequency cepstral coefficients (MFCCs) and hidden Markov models (HMMs) support the finding that a reduction in training data by a factor of 10 does not significantly affect the recognition performance.

## Introduction

**Figure 1:**
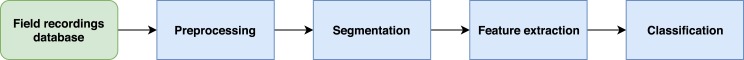
A four-step workflow summarizes the automated bird recognition process.

Automated acoustic species recognition and event detection are becoming increasingly popular instruments for supporting biodiversity conservation (Zhao et al., 2017). The main reason for this popularity is that automation allows scalable monitoring and unattended data collection simultaneously at multiple study sites. The automated recognition of bird vocalizations follows a typical analysis workflow and includes four major steps: signal preprocessing, audio segmentation, feature extraction, and classification (Fig. 1) (Zhao et al., 2017).

 The audio preprocessing step consists of resampling and noise suppression. This step facilitates the subsequent audio feature extraction and data classification stages. Next, audio segmentation is performed to select portions of the signal considered promising and to eliminate silent segments or segments with predominantly background noise. The feature extraction steps provide a compact description of the acoustic events of interest available in the soundscape recording. For feature extraction, the raw signal is transformed to a small set of representative audio features that preserve the information of interest and discard variability due to unknown or unwanted sources (e.g., environmental sounds and equipment setup). Finally, in the classification step, the decision is made based on the feature vectors and precomputed models.

There are two key aspects of automated acoustic monitoring: audio data representation and machine learning (Jing et al., 2016). Here, we focus on the former because it has a profound impact on the computational effort and memory required for implementing automated species and event recognition tasks.

In this paper, audio data are represented by Mel-frequency cepstral coefficients (MFCCs) (Aparna, 2015; Albornoz et al., 2017; Stastny, Munk & Juranek, 2018), which are computed for short segments of the audio signal. MFCCs have acceptable average classification accuracy with different machine learning classifications (Oo, 2018). This approach can be thought of as the transformation of a soundscape recording into a numeric matrix of audio features, where rows represent temporal frames and columns represent cepstral coefficients. The Mel-frequency scale is an approximation of the human auditory system, with the use of filter banks. Based on this scale, the coefficients are a cepstrum representation of a time-windowed signal. The coefficients are derived after applying a discrete Fourier transform to nonlinear frequency scales. To capture the temporal dynamics of the soundscape, the MFCCs are typically supplemented with their first and second temporal derivatives, and this process results in a multidimensional feature vector. Because the size of the feature matrix depends linearly on the length of the soundscape recordings, this matrix can include a large number of rows; thus, significant time and memory resources are required for both model creation and signal classification.

There are various approaches that might assist in coping with the problem of an ungainly feature matrix. A trivial approach for feature matrix reduction would be to reduce the overlap between successive short-term segments of the signal for which the audio features are computed. However, this also means a reduction in the time resolution of feature extraction; thus, this approach might increase the risk of omitting important events, which would reduce the accuracy of temporal boundary detection. In addition, in some cases, depending on the duration of the stationary state of the sound events, it might be feasible to select a larger window for the audio frame analysis. However, a larger window size might smear event onsets and transitions between events, which would cause a loss of information. Furthermore, a larger window might capture more than one acoustic event and combine their spectra, which would reduce the fidelity of audio features.

Nontrivial solutions require additional effort for signal preprocessing, feature matrix postprocessing, or both. Soundscape preprocessing aims to select interesting segments of the signal before computing the audio features. Potamitis (2014) used a morphological image processing operator to select important regions in a spectrogram of bird vocalizations; then, the MFCCs were computed only for those parts. In contrast, postprocessing aims to reduce data after the audio feature matrix is computed. Data can be reduced in two ways. First, the number of columns can be reduced; this involves feature ranking and selection or feature space transformation. Second, the number of rows can be reduced; this requires the use of data representation or data reduction methods.

Numerous studies have developed methods for feature selection, transformation, and projection to save time and improve accuracy in the machine learning stage. For various speech and speaker recognition tasks, principal component analysis (Babu, Anitha & Anjana, 2013; Quan et al., 2013; Ahmad et al., 2015; Sharma, Kumar & Das, 2015; Kumar, Katti & Das, 2016; Charan et al., 2017), linear discriminant analysis (Sharma, Kumar & Das, 2015; Souissi & Cherif, 2015), stochastic neighbor embedding (t-SNE) (Charan et al., 2017), naive dropping, F-ratio (Sharma, Kumar & Das, 2015), and other methods have been shown to provide advantageous results.

Data representation methods, such as sampling, have been discussed for signal processing (Masry, 1971; Jerri, 1977; Yen, 1997). Lv, Liu & Yang (2013) showed the effects of different data sampling methods on an engineering benchmark modeling problem. A similar approach was used for speech and music signals (Zarmehi, Shahsavari & Marvasti, 2017) and voice signals (Mashhadi et al., 2017). Stowell & Plumbley (2014) tested the temporal summarization method on bird vocalizations.

Data sampling, data subset selection, and time-series representation methods are seldom used in the context of bioacoustics studies because annotated data are often lacking for model creation. Reducing the number of data points (Fu, 2011) has been used primarily to facilitate data visualization. Here, we need to point out that the abovementioned trivial approach for reducing the size of a feature matrix (i.e., by reducing the overlap between successive audio segments) is equivalent to resampling the rows in the feature matrix. A previous study showed that randomly sampling the feature matrix, preserving a subset of rows, was successful in the creation of a Gaussian mixture model-universal background model in the context of speaker recognition (Fauve et al., 2007). Recently, temporal feature aggregation has also been employed for that purpose (Nelus, Gergen & Martin, 2017).

In the present work, a data reduction step was added as an additional postprocessing step after feature extraction in the context of a bird recognition task. Furthermore, we investigated the applicability of three data reduction methods and evaluated their capacity to reduce the number of rows in the feature matrix used for model creation without a significant reduction in the bird classification performance. In ‘Materials & Methods’, we first outline the contexts for using data reduction methods; then, we summarize the rationale for each method and outline the experimental protocol we used for a comparative evaluation of these data reduction methods. In ‘Results & Discussion’, we discuss some differences in recognition accuracy and data processing speed. The experimental results support the notion that a significant reduction in computational time during the training stage is achievable without a notable loss of recognition accuracy.

## Materials & Methods

### Automated bird classification

**Figure 2:**
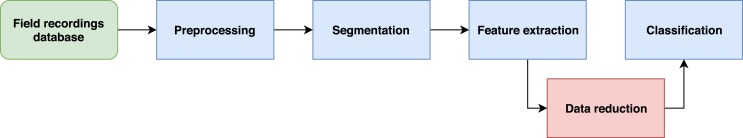
The proposed five-step workflow for speeding up the training of automated bird recognition models.

When abundant training data are available for robust modeling, a data subset creation method can be employed to reduce the size of the representative training dataset. This approach can speed up the convergence of the training process and significantly speed up model creation. In the current research, we focus on reducing the number of feature vectors in the feature matrix used for model creation by means of a data reduction method. For that purpose, we inserted an additional fifth stage into the data processing workflow (Fig. 2) that represents postprocessing of the feature matrix. The use of a more compact feature matrix is expected to reduce the time necessary for model creation and achieve better performance during classification. This hypothesis was based on the notion that data reduction methods could highlight fundamental characteristics of the feature series (Esling & Agon, 2012), which, in our case, were represented as rows in the feature matrix.

As different data reduction methods rely on different assumptions, their performance depends on the inherent characteristics of the data series being processed. In the present study, we investigate the applicability of three data reduction methods: (1) random sampling (RS), (2) uniform sampling (US), and (3) piecewise aggregate approximation (PAA); we employ these methods in a comparative evaluation. Their performance is evaluated with respect to the processing time, precision, and accuracy achieved in the bird recognition task at different levels of data reduction. We investigated the differences between the accuracy achieved and the amount of data processed when retaining different subsets of the initial dataset.

### Data reduction methods

#### Random sampling

In the RS method, the initial feature matrix with *n* rows is reduced to a new matrix that consists of *N* rows, where *N* is a preselected number and *n* ≥ *N*. The number of columns is preserved. The new feature matrix consists of randomly selected subsets of feature vectors taken from the initial feature matrix. Briefly, the initial dataset is first enumerated; then, the feature vectors to be retained for the new subset are selected in a random manner. This is implemented by a random number generator with a uniform distribution, which generates nonrepeating integer values. Here, the RS method is considered the baseline against which the other methods are evaluated.

#### Uniform sampling

The US method (Åstrom, 1969) is the simplest and most frequently used data reduction method. In this method, *N* feature vectors are taken out of the initial feature matrix at equal spacing *h*. The sampling interval, *h*, controlled the data reduction rate, given by the ratio *n/N*, where *n* and *N* are the numbers of rows in the initial and the new feature matrices, respectively.

#### Piecewise aggregate approximation

The PAA method (Keogh et al., 2001) reduces the feature matrix with *n* rows to a new feature matrix with *N* rows, where *n* ≥* N* and *N* is a factor of *n* (Eq. (1)). Here, the initial feature matrix is first segmented into *N* equal-sized sections. Then the mean value of the feature vectors within each section are calculated to give *N* new feature vectors (Keogh et al., 2001).

(1) \begin{eqnarray*}{\overline{x}}_{i}= \frac{N}{n} \sum _{j= \frac{n}{N} \left( i-1 \right) +1}^{ \frac{n}{N} i}{x}_{j}.\end{eqnarray*}

### Experimental protocol

In the current study, we made use of real field recordings of an avian lapwing species, *Vanellus chilensis*, previously described by Ganchev et al. (2015) and Ventura et al. (2015). *Vanellus chilensis* vocalizes from the ground and in flight, particularly during courtship and territory displays as well as in response to the presence of potential predators. The most often heard vocalization is a strident “keh-keh-keh-keh-keh...” that is consistently repeated by alarmed birds (Gwynne et al., 2010). The audio dataset contained 90 recordings, which comprised approximately 45 min of target sounds. Among these, 38 recordings were virtually clean of competing interference (∼18 min), and 52 recordings contained other competitive low-volume background sounds that overlapped with the target signals (∼24 min). These recordings were used to train the *V. chilensis* (VACH) model.

In addition, a number of soundscape recordings (in .wav format) were provided, with durations of 14–30 min, sampled at 48 kHz, with a resolution of 16 bits per sample. These recordings were used to develop an acoustic background (BG) model containing sounds of the environment, such as birds and other animals. In fact, the background dataset was created by combining two datasets of audio recordings that were representative of the Pantanal soundscapes. The first dataset consisted of 54 14-min recordings with a total duration of over 12 h. The second dataset contained 32 30-minute recordings, which comprised approximately 15 h of continuous recordings from a sound recording station located in a forest. This latter habitat was typically avoided by the target species; thus, the recordings from that station were regarded as free of *V. chilensis* sounds, although occasionally some vocalizing lapwings may have flown over the site. Thus, approximately 27 h of audio recordings from the Pantanal were available for training the acoustic BG model.

We created an additional model that represents a community of 40 other bird species present in the Pantanal. This dataset (OTHER) consisted of 1532 audio segments of vocalizations from birds other than *V. chilensis*. These data are a subset of the BirdCLEF 2015 dataset.

Finally, we used a test dataset with 14 soundscape 14-min recordings to evaluate the experiments. These 14-min recordings were not processed or edited manually and contained competing sounds from multiple species and some interference of abiotic origin. Both the training and test datasets are available at the CO.BRA website <http://cobra.ic.ufmt.br/?page_id=161 >.

We extracted the MFCC audio features from all audio datasets, as described in Ganchev (2011). The preprocessing and segmentation steps were performed as described by De Oliveira et al. (2015). In brief, audio data were downsampled from 48 kHz to 24 kHz in the preprocessing stage, and a morphological image operator was employed for the segmentation technique.

We computed 16 MFCCs with a sliding window of 20.0 ms and a skip step of 5.0 ms. We then computed the first and second temporal derivatives (delta and delta-delta coefficients) of the MFCCs, as described by Dufour et al. (2013). This resulted in a feature vector of 48 parameters. The matrix formed by all audio feature vectors computed from the initial dataset was then postprocessed with a data reduction method, which eliminated some portion of the feature vectors. Here, we employed three data reduction methods at four data reduction rates, which retained 5%, 10%, 20%, and 40% of the initial 13 638 950 feature vectors. Thus, we analyzed a total of 12 different subsets of feature vectors as the training dataset.

To perform comparative evaluations, we used the HMM-based classifier described in De Oliveira et al. (2015). An HMM was selected for birdsong modeling due to its ability to make use of grammar, i.e., prior knowledge of the problem. This is especially convenient, as our long-term research project^1^ aims to achieve the creation of statistical models 1Project INAU II, URL: http://cobra.ic.ufmt.br/.of the sound emissions of multiple bird species and their song types. The use of grammar and the implicit assumption made that the next state depends only on the previous state leads to a significant reduction in the amount of training data required for robust model creation. Other classifiers, such as convolutional neural networks (CNNs), for instance, have shown superior accuracy in many applications (Lasseck, 2018), yet this is usually at the cost of a larger amount of computation during training. CNNs also require larger training datasets or additional effort for data augmentation (Salamon & Bello, 2017), as these classifiers do not make assumptions about the temporal structure of birdsongs but learn it directly from the training dataset.

In short, based on the Hidden Markov Model Toolkit (Young et al., 2006), we developed a classifier with a 3-state HMM and 48 mixture components. The training was performed with 80 iterations of the Baum–Welch algorithm.

To evaluate the performance of the three data reduction methods, we measured the processing time required and the recognition in terms of two performance metrics—precision and accuracy, measured in percentages at the frame level against NO VACH frames (OTHER or BG).

We measured the time required for selecting the different data subsets and for training the models with these subsets to evaluate the value of including the data reduction stage in the workflow. All tests were carried out on a computer with an operational memory of 64 GB and eight-core Intel Xeon E5-2660 processors, which operated at 2.6 GHz with 25 MB of cache.

This study is part of the following biodiversity monitoring project: Sounds of the Pantanal—The Pantanal Automated Acoustic Biodiversity Monitoring of INAU, Cuiabá, Mato Grosso, Brazil, conducted under SISBIO permit no. 39095 (KLS).

## Results & Discussion

**Figure 3:**
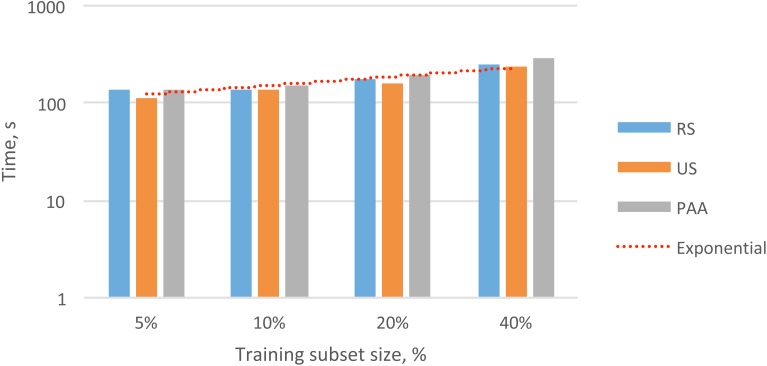
Overall exponential trend and total processing time for selecting different subsets of audio feature vectors (5–40%) with different data reduction methods.

To evaluate the practical value of reducing the data in the audio feature matrix, we measured the processing time required for each of the methods described (i.e., RS, US, and PAA). In Table 1, we present the processing time needed to obtain the different subsets of the feature vectors. These training sets contained 5%, 10%, 20%, and 40% of the initial dataset. In Fig. 3, we present the total times required for data reduction and the overall trend.

**Table 1:** Total processing time required for obtaining different subsets of data that comprised 5%, 10%, 20%, or 40% of the initial dataset.

Method	5%	10%	20%	40%
RS	2 min 14 s	2 min 16 s	2 min 57 s	4 min 08 s
US	1 min 53 s	2 min 20 s	2 min 42 s	3 min 56 s
PAA	2 min 18 s	2 min 30 s	3 min 11 s	4 min 50 s

In nearly all cases, the fastest method for selecting subsets of data was US, followed by RS and then PAA. This result was probably due to the higher complexity of the RS and PAA methods compared to that of the US method. In particular, the RS method required additional time to generate a sequence of unique random numbers with a uniform distribution. The PAA method required additional time to compute the average values for each section to create subsequent feature vectors.

Datasets of the same size contained different selections of feature vectors (due to the different data reduction methods). Next, we were interested in determining whether the different datasets generated significant differences in the recognition performance and convergence speed during HMM training. For that purpose, we performed 12 experiments, each with a different data subset, and each experiment was used to train three different HMMs: (i) the VACH model, (ii) the BG model, and (iii) the OTHER model. In Table 2, we show the total times required for model training. In Tables 3 and 4, we show the precision and accuracy obtained with different feature subsets for the VACH detection task. We evaluated the time required for convergence of the training process and the classification accuracy to determine which of the three methods might be most appropriate for practical applications.

**Table 2:** Total times for training the HMMs with different subsets of audio features.

Method	5%	10%	20%	40%
RS	1 h 25 min	5 h 58 min	33 h 10 min	164 h 04 min
US	1 h 36 min	7 h 09 min	37 h 28 min	290 h 10 min
PAA	1 h 43 min	7 h 37 min	41 h 47 min	303 h 10 min

**Table 3:** Performance in terms of the precision of classification for different subsets of audio feature vectors.

Method	5%	10%	20%	40%	…	Baseline
RS	56.2%	58.4%	53.1%	47.8%		64.1%
US	65.6%	65.8%	65.3%	64.6%		64.1%
PAA	0.0%	0.0%	63.3%	64.7%		64.1%

**Table 4:** Performance in terms of the classification accuracy for different subsets of audio feature vectors.

Method	5%	10%	20%	40%	…	Baseline
RS	96.3%	96.7%	96.2%	95.4%		97.5%
US	97.6%	97.7%	97.6%	97.6%		97.5%
PAA	95.8%	95.8%	97.2%	97.6%		97.5%

**Figure 4:**
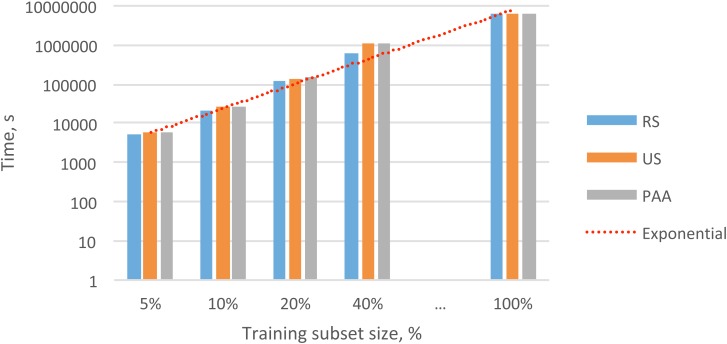
Overall exponential trend and total times for training the HMMs with different subsets.

As shown in Table 2 and Fig. 4, the datasets created with different data reduction methods actually led to dissimilar training times for the HMMs. In all cases, the datasets built with the RS method led to the fastest convergence in HMM training. In analyzing the results, we observed that the training iterations were stopped much earlier for the RS datasets than for the other datasets due to the lack of model improvement. The datasets built with the PAA method led to the slowest convergence during HMM training. This result suggested that averaging the feature vectors might have smeared some important variability among the feature vectors that was needed for VACH recognition.

We found that the smallest datasets selected by the PAA method (i.e., 5% and 10% of the initial data; Tables 3 and 4) were insufficient for training a robust HMM. This failure occurred mostly because the PAA method had to average many vectors to produce these small data subsets. This averaging eliminated important details and variability in the feature vectors; thus, the specific features that distinguished the three classes (VACH, BG, and OTHER) were lost. The accuracy of RS was affected by the randomness in each subset, where just a few relevant frames were selected in each subset.

An analysis of the results shown in Tables 3 and 4 led to the conclusion that the US method performed best. The models trained with the US subsets even performed well for the smallest subset, which held only 5% of the original data. The predictable recognition performance and the low complexity of this method made it preferable to the other data reduction methods for the *V. chilensis* detection setup.

It took approximately 1767 h to train the HMM models with the initial dataset, which contained 13 638 950 audio feature vectors. This training time was considered a reference for comparing the training times required after applying the data reduction methods. When we combined the cumulative times for performing data reduction with the US method (Table 1) and the times for HMM training (Table 2), we observed a dramatic reduction in the total processing time relative to the reference (Table 5).

**Table 5:** Percentage reduction of the overall training times after applying the indicated data reduction methods, compared to the time needed to train a model with the initial dataset.

Method	5%	10%	20%	40%
RS	99.9%	99.7%	98.1%	90.7%
US	99.9%	99.6%	97.9%	83.6%
PAA	99.9%	99.6%	97.6%	82.8%

Depending on the size of the audio feature subset, we obtained relative reductions in training times of 83.6 to 99.9%, without any decline in recognition performance. When we evaluated the results for all methods, we found that the dataset with 10% of the audio feature vectors selected by the US method provided the best trade-off among the data reduction rate, time required for model training, and recognition performance. This method reduced the HMM training time by 99.6% and preserved recognition precision and accuracy.

## Conclusions

We introduced an additional postprocessing step for data reduction to reduce the time required for training the HMMs in the *V. chilensis* recognition task. We found that the US method was the most advantageous when compared to the other two methods; it provided a 99.6% relative reduction in training time with no loss of accuracy or precision. Based on the experimental results, we conclude that data reduction methods improve the efficiency of soundscape processing. For instance, these methods might be used for preliminary scans of large datasets of recordings (e.g., annual cycles of continuous soundscape recordings) to spot specific types of acoustic activity.

## Additional Information and Declarations

### Data Availability

The following information was supplied regarding data availability: All data based on our study is available at the website of the Computational Bioacoustics Research Unit, Federal University of Mato Grosso (UFMT):
http://cobra.ic.ufmt.br—Data Sets—Oliveira 2020. Code to apply data reduction is available at https://github.com/thiventura/representation.
